# Marine Sulfated Polysaccharide PMGS Synergizes with Paclitaxel in Inhibiting Cervical Cancer In Vitro

**DOI:** 10.3390/md21050259

**Published:** 2023-04-23

**Authors:** Xuan Xia, Yanhong Wang, Yingchun Shao, Jiazhen Xu, Bing Liang, Wenjing Liu, Jun Zeng, Chunxia Li, Huashi Guan, Shixin Wang, Dongming Xing

**Affiliations:** 1 Key Laboratory of Marine Drugs of Ministry of Education, and Shandong Provincial Key Laboratory of Glycoscience and Glycotechnology, School of Medicine and Pharmacy, Ocean University of China, Qingdao 266003, China; 2 Marine Biomedical Research Institute of Qingdao, Qingdao 266071, China; 3 Qingdao Cancer Institute, Qingdao University, Qingdao 266071, China; 4 School of Life Sciences, Tsinghua University, Beijing 100084, China

**Keywords:** marine sulfated polysaccharides, paclitaxel, cervical cancer, synergistic effect

## Abstract

The incidence and mortality of cervical cancer in female malignancies are second only to breast cancer, which brings a heavy health and economic toll worldwide. Paclitaxel (PTX)-based regimens are the first-class choice; however, severe side effects, poor therapeutic effects, and difficulty in effectively preventing tumor recurrence or metastasis are unavoidable. Therefore, it is necessary to explore effective therapeutic interventions for cervical cancer. Our previous studies have shown that PMGS, a marine sulfated polysaccharide, exhibits promising anti-human papillomavirus (anti-HPV) effects through multiple molecular mechanisms. In this article, a continuous study identified that PMGS, as a novel sensitizer, combined with PTX exerted synergistic anti-tumor effects on cervical cancer associated with HPV in vitro. Both PMGS and PTX inhibited the proliferation of cervical cancer cells, and the combination of PMGS with PTX displayed significant synergistic effects on Hela cells. Mechanistically, PMGS synergizes with PTX by enhancing cytotoxicity, inducing cell apoptosis and inhibiting cell migration in Hela cells. Collectively, the combination of PTX and PMGS potentially provides a novel therapeutic strategy for cervical cancer.

## 1. Introduction

Cervical cancer is one of the most common malignant tumors in women. In 2020, cervical cancer was the fourth most commonly diagnosed cancer and the fourth leading cause of cancer death among women, with an estimated 604,000 new cases and 342,000 deaths worldwide [1]. Notably, the incidence and mortality of cervical cancer in gynecological malignancies are second only to breast cancer globally [1], which is consistent with the epidemiological situation in China [2]. The current first-class therapy regimens for cervical cancer are surgery, radiotherapy, and chemotherapy [3,4]. Chemotherapy combined with surgery and radiotherapy is used to treat advanced and recurrent cervical cancer [3,4]. Early diagnosis and therapy for cervical cancer result in a reasonable prognosis. However, the 5-year survival rates for advanced cervical cancer range from 16 to 35% for stage III-IVA, and the prognosis is still poor for recurrent and metastatic cervical cancer [3,5]. Hence, it is urgently needed to develop novel efficient therapeutic strategies to increase long-term survival and reduce recurrence and metastasis in cervical cancer therapy.

As is well-known, cervical cancer is transformed from cervical intraepithelial neoplasia (CIN) caused by prolonged high-risk human papillomavirus (HPV) infection [6]. The E6 and E7 proteins that exist in the early transcriptional coding of HPV are the main factors leading to cervical cancer by promoting the degradation of the tumor suppressor protein P53 and leading to the premature transition of cells from the G1 phase to the S phase [7,8,9]. If the immune system is unable to remove the cancerous cells and clear the viral infection in time, cervical cancer may occur [7,8,9]. Thus, the prevention and treatment strategy of cervical cancer can be as follows: block the virus infection; eliminate the virus infection; down-regulate the E6 and E7 proteins; enhance immunity; inhibit tumor cell proliferation, migration and invasion, etc.

Our previous studies have found that polymannuroguluronate sulfate (PMGS), which is a sulfated polysaccharide synthesized from marine alginate derived from brown seaweed [10,11], possesses robust anti-HPV activities in vitro and in vivo, alleviates leukopenia caused by chemotherapy, promotes the differentiation and maturation of CD4^+^ T cells in the thymus, inhibits the apoptosis of CD4^+^ T cells [10], and inhibits the expressions of E6 and E7 genes [11]. The results suggest the potential application of PMGS in the prevention and treatment of cervical cancer within the foreseeable future.

Moreover, studies have shown that marine polysaccharides may play an anti-tumor role by inducing tumor cell apoptosis, inhibiting the formation of tumor blood vessels, and activating the immune system [12,13,14]. The mechanism of action is different from that of chemotherapy drugs for cervical cancer, so there is a possibility of synergism and complementarity for polysaccharides combined with the chemotherapy drugs used in the treatment of cancer.

Paclitaxel (PTX) is a natural compound extracted from the bark of the genus Sequoia in the sequoia family [15]. As a chemotherapy drug, PTX is extensively used to treat several cancers as a first- or second-line treatment option, especially cervical, breast, and other cancers with high incidence rates [16,17]. Unfortunately, high doses of mono-drugs are prone to side effects, patients are susceptible to developing drug resistance, and it is difficult to effectively prevent tumor recurrence or metastasis [18,19]. Therefore, it is urgent to explore more effective therapeutic methods with a lower toxicity for chemotherapy.

In this study, we evaluated whether the marine sulfate polysaccharide PMGS combined with PTX enhances the antineoplastic activity of PTX in cervical cancer cells and further explored the underlying molecular mechanisms. Our results show that subtoxic concentrations of PMGS synergistically promoted PTX-induced tumor growth and metastasis repression in cervical cancer cells by inducing cell apoptosis and inhibiting cell migration, indicating that PMGS may potentially be a novel sensitizer that synergizes with PTX in blocking cervical cancer.

## 2. Results

### 2.1. Cytotoxic Activity of PMGS and PTX in Cervical Cancer Cells

**Figure 1:**
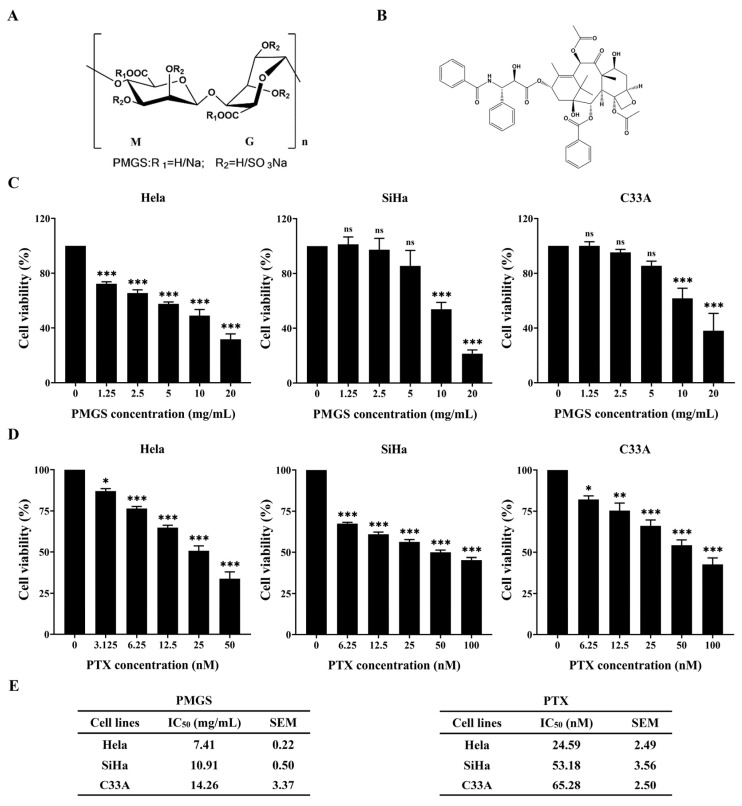
Cytotoxic activity analysis of PMGS and PTX in cervical cancer cells. (**A**) Chemical structure of PMGS. (**B**) Chemical structure of PTX. (**C**,**D**) CCK-8 assay was performed to detect cell viability in Hela, SiHa, and C33A cells 48 h after cells were treated with PMGS (**C**) or PTX (D). (**E**) The IC_50_ values of PMGS and PTX in Hela, SiHa, and C33A cells. Error bars, SEM. ns, not significant; * *p* < 0.05; ** *p* < 0.01; *** *p* < 0.001.

Since high-risk subtypes of HPV are the leading cause of cervical cancer, and HPV-18 and HPV-16 are the two main high-risk subtypes of HPV [3,20], we assessed the drug effects on HPV-18-positive Hela cells, HPV-16-positive SiHa cells, and HPV-negative C33A cells. We first assessed the cytotoxic effects of PMGS and PTX, and the results show that both drugs decreased cell viability in a dose-dependent way (Figure 1C,D). The IC_50_ values of PMGS in the Hela, SiHa, and C33A cells were 7.41 mg/mL, 10.91 mg/mL, and 14.26 mg/mL, respectively (Figure 1E). The IC_50_ values of PTX were 24.59 nM, 53.18 nM, and 65.28 nM, respectively (Figure 1E). The results revealed that both PMGS and PTX showed more robust proliferation inhibition in the Hela cells than in the SiHa and C33A cells (Figure 1C–E). This indicates that HPV-18-transformed Hela cells could be more sensitive to PMGS and PTX. This might be due to the HPV strain being a susceptible factor or the influence of the cell type itself, which required further investigation.

### 2.2. The Interaction of PMGS and PTX in Cervical Cancer Cells

**Figure 2:**
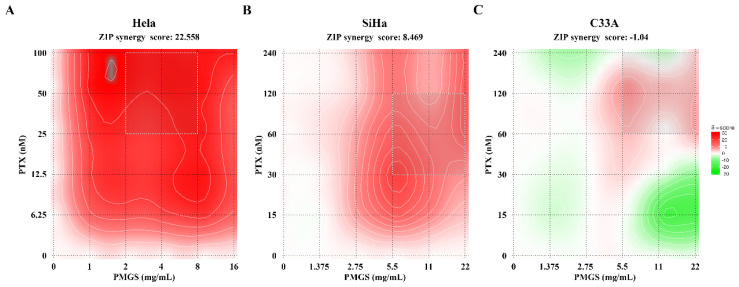
The interaction analysis of PMGS and PTX in cervical cancer cells. (**A**–**C**) Heatmaps of the combination drug responses of PMGS and PTX in Hela (**A**), SiHa (**B**), and C33A (**C**) cells. Cell viability was detected using a CCK-8 assay 48 h after cells were treated with PMGS and PTX, and then the online SynergyFinder software was used to estimate the drug interaction using ZIP synergy scores. ZIP synergy scores < −10 indicated antagonism, scores from −10 to 10 suggested addition, and scores > 10 showed synergism.

Side effects restrain the application of chemotherapies in clinic. Therefore, to improve drug efficacy, as well as reduce adverse effects, we tested whether PMGS could synergize with PTX in the treatment of cervical cancer. According to the IC50 values of PMGS and PTX, we designed a dose-range experiment and detected cell viability, in which dose-escalating PMGS (0–22 mg/mL) and PTX (0–240 nM) were added to Hela, SiHa, and C33A cells alone or in combination (Supplementary Tables S2–S4). The online SynergyFinder software was then used to calculate the ZIP synergy scores based on the viability index (Figure 2). The results show that the average drug interaction scores were 22.558 in the Hela cells, 8.469 in the SiHa cells, and −1.04 in the C33A cells. Collectively, the combination of PMGS and PTX displayed significant synergistic effects in the Hela cells rather than in the SiHa and C33A cells (ZIP synergy scores > 10).

### 2.3. PMGS Synergistically Promoted PTX-Induced Cytotoxic Effect in Hela Cells

**Figure 3:**
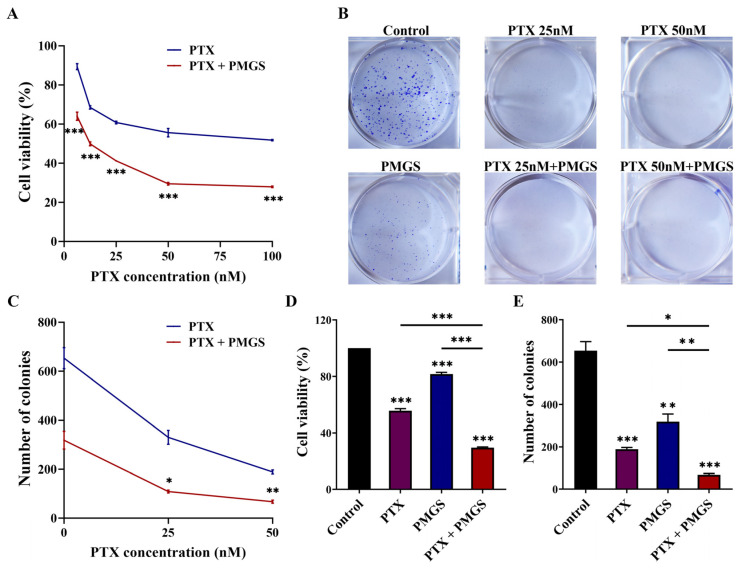
PMGS synergistically promoted PTX-induced cytotoxic effect in Hela cells. (**A**–**C**) Under different treatments with either PMGS or PTX alone or in combination, cell viability was detected using a CCK-8 assay (**A**), and colony formation capacity was detected using a colony formation assay (**B**,**C**) in Hela cells. (**D**–**E**) Cell viability (**D**) and colony formation (**E**) were detected after treatment with either 2 mg/mL PMGS or 50 nM PTX alone or in combination in Hela cells. Error bars, SEM. * *p* < 0.05; ** *p* < 0.01; *** *p* < 0.001.

In addition, we continued to explore the synergistic effect of PMGS combined with PTX on Hela cells. As shown in Figure 2, the white rectangle suggests the strongest synergy area. The result indicates that 2 mg/mL was the lowest concentration of PMGS required to achieve maximum synergy. Hence, 2 mg/mL PMGS (inhibition ratio: 18.33% ± 1.23%) was selected as the optimal concentration in the following experiments. To determine the appropriate concentration of PTX, cell viability (Figure 3A) and colony formation (Figure 3B,C) were evaluated after the treatment of PMGS or PTX alone or in combination. The results suggest that the concentration of 50 nM was the optimal dose of PTX required to achieve the highest synergistic effect (Figure 3A–C). After the different treatments, 2 mg/mL PMGS or 50 nM PTX alone reduced cell viability and colony formation, and the effects were further decreased by the combination of PMGS and PTX (Figure 3D,E). Altogether, PMGS with a much lower concentration than IC_50_ synergistically improved the cytotoxicity of PTX against the Hela cells.

### 2.4. The Combination of PMGS and PTX Induced Cell Cycle Arrest and Apoptosis in Hela Cells

**Figure 4:**
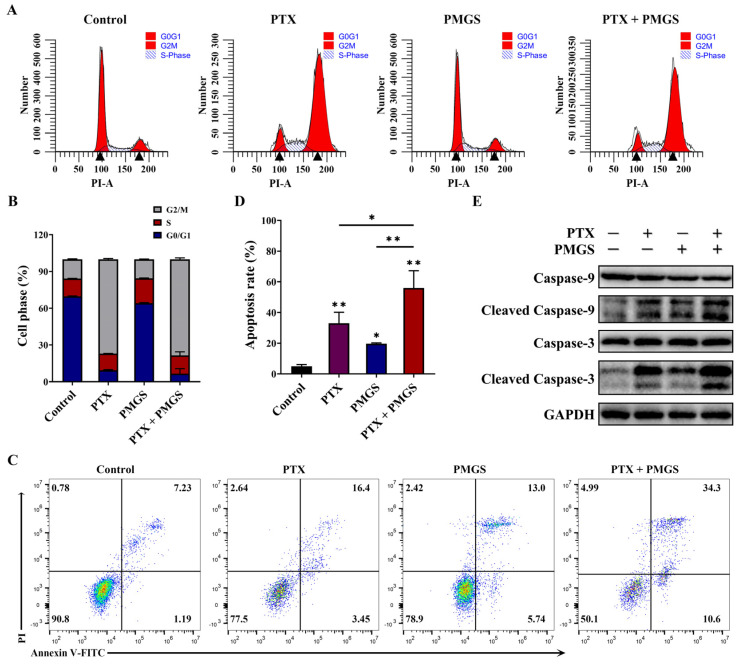
The combination of PMGS and PTX induced cell cycle arrest and apoptosis in Hela cells. (**A**,**B**) Cell cycle was detected using a PI staining assay, (**C**,**D**) cell apoptosis was detected using an Annexin V-FITC/PI assay, (**E**) the expressions of apoptosis-related proteins were detected using Western blot after treatment with either PMGS or PTX alone or in combination in Hela cells. GAPDH was used as a loading control (**E**). Error bars, SEM. * *p* < 0.05; ** *p* < 0.01.

To investigate the mechanisms of the synergistic effect of PMGS in combination with PTX on Hela cells, a PI staining assay using flow cytometry was used to analyze the cell cycle distribution. As shown in Figure 4A,B, PTX alone arrested the cell cycle at the G2/M phase in the Hela cells. However, PMGS neither participated in the regulation of the cell cycle nor affected the PTX-mediated cell cycle arrest in the Hela cells. Meanwhile, the drug combination blocked the cell cycle at the G2/M phase in the Hela cells, similar to PTX treatment alone (Figure 4A,B).

An Annexin V-FITC/PI staining assay was also performed to detect cell apoptosis. The results show that PMGS or PTX alone induced cell apoptosis to a certain extent, while PMGS combined with PTX further promoted apoptosis in the Hela cells (Figure 4C,D).

To confirm that PMGS enhanced PTX-induced apoptosis in the Hela cells, we analyzed the expressions of apoptosis-related proteins using Western blot. As shown in Figure 4E, PMGS or PTX alone induced both caspase-9 and caspase-3 cleavage, and the drug combination further raised the cleavage. Taken together, the combination of PMGS with PTX inhibited the cell growth of the Hela cells by activating cell apoptosis.

### 2.5. PMGS in Combination with PTX Inhibited Migration and Invasion in Hela Cells

**Figure 5:**
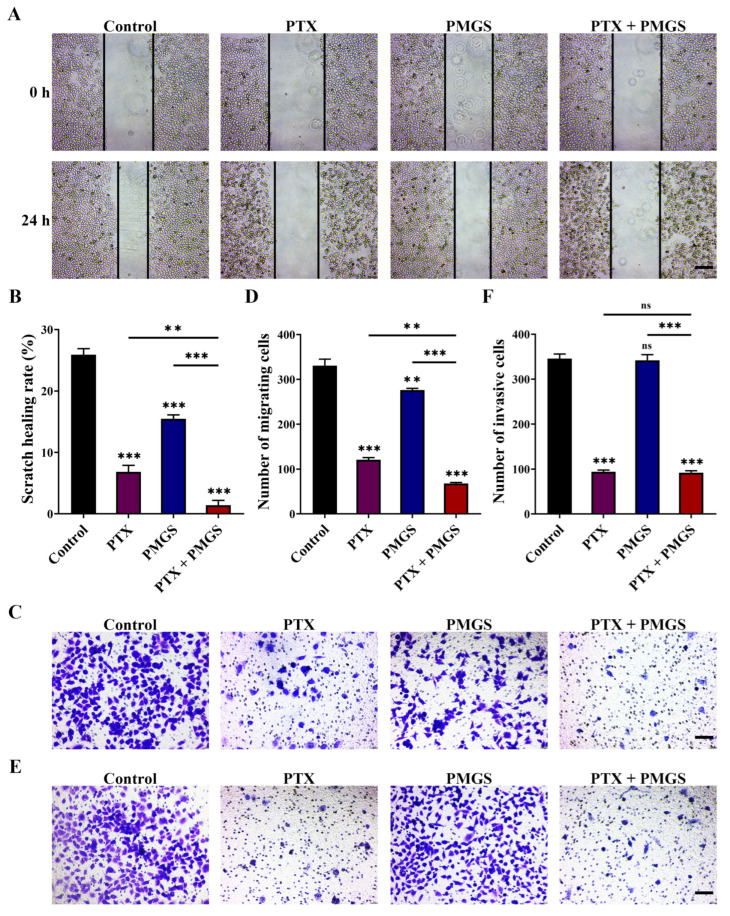
PMGS in combination with PTX inhibited migration and invasion in Hela cells. (**A**–**D**) Cell migration was detected using wound-healing assay (**A**,**B**) and Transwell migration assay (**C**,**D**) after treatment with either PMGS or PTX alone or in combination for 24 h and 48 h in Hela cells, respectively. (**E**,**F**) Cell invasion was detected using Transwell invasion assay after 72 h with the treatment of PMGS or PTX alone or in combination in Hela cells. Magnification, ×100; scale bars, 100 μm. Error bars, SEM. ns, not significant; ** *p* < 0.01; *** *p* < 0.001.

Since metastasis is the leading cause of cervical cancer, we also investigated the effect of PMGS in combination with PTX on the migration and invasion of Hela cells. The data show that PMGS or PTX alone delayed scratch wound healing compared with the control, and PMGS combined with PTX further accelerated scratch healing compared with the other three groups (Figure 5A,B). Correspondingly, a Transwell migration assay suggested that PMGS in combination with PTX synergistically inhibited the migration of the Hela cells (Figure 5C,D).

Furthermore, as shown in Figure 5E,F, the Transwell invasion assay results indicated that PTX alone blocked cell invasion. However, PMGS was not involved in cell invasion, nor did it influence the PTX-induced invasion of the Hela cells. In summary, the drug combination inhibited the invasion of the Hela cells in a similar manner to the effect of PTX alone (Figure 5E,F).

## 3. Discussion

Paclitaxel, along with cisplatin and doxorubicin, is the most common chemotherapeutic drug in cancer therapy [16,17]. Paclitaxel plus cisplatin (TP) or carboplatin (TC) is the standard treatment for cervical cancer [21,22]. However, the long-term repeated and high dose of PTX-based treatment leads to severe toxicity and drug resistance [9,10]. It is additionally tricky to effectively prevent tumor recurrence or metastasis [9,10]. Therefore, it is necessary to explore an effective therapeutic method for cervical cancer. Our study shows that PMGS, a marine sulfate polysaccharide, improved anti-tumor efficacy as a new combination with PTX in cervical cancer for the first time. Marine sulfated polysaccharides have been extensively applied in many aspects, such as in anti-tumor, anti-viral, anti-inflammatory, anticoagulant, antimicrobial, and antilipemic agents and in the therapy of regenerative medicine [23,24]. PMGS in our study is a polysaccharide polymannuroguluronate sulfate, in which the initial reactant is derived from brown seaweed [10,11]. Previous studies have demonstrated that PMGS ameliorates leukopenia by inhibiting CD4^+^ T-cell apoptosis [10] and plays critical roles in repressing HPV [11]. Our study further exhibits the anti-tumor effects of PMGS as a sensitizer combined with PTX in cervical cancer cells, which has not been reported before.

The results of the drug interaction analysis showed that PMGS combined with PTX had synergistic effects on Hela cells (SynergyFinder) (Figure 2A), while the combination played an additive role in SiHa and C33A cells (Figure 2B,C). Hela, SiHa, and C33A cells are HPV-18-positive, HPV-16-positive, and HPV-negative cells, respectively, indicating that PMGS might act preferentially and selectively on HPV18-transformed Hela cells, which require further investigation. Additionally, the synergistic effects of PMGS combined with PTX on Hela cells may indicate a more accurate targeted therapy for patients with cervical cancer who are infected with HPV-18, which also needs further exploration.

Notably, PMGS alone was also demonstrated to play an anti-tumor role in cervical cancer cells, except for the combination of PMGS and PTX. Our study shows that PMGS with a concentration well below IC_50_ induced the cytotoxicity, apoptosis, and migration of Hela cells. However, it did not affect the cell cycle and invasion of Hela cells. Both migration and invasion are malignant behaviors of tumor cells with correlations and differences. Migration is a prerequisite to invasion in which invasion cannot be achieved without migration, but migration can be completed without invasion [25,26]. Our results indicate that PMGS had no effects on the adhesion and proteolysis of the extracellular matrix in Hela cells, which requires further research. Additionally, PMGS also did not affect PTX-induced invasion or the cell cycle, so PMGS combined with PTX still decreased invasion and induced cell cycle arrest in Hela cells.

In this article, we conducted a preliminary study of the synergistic effects of the combination of PMGS and PTX on Hela cells, and the follow-up mechanisms need further investigation, such as the mechanisms of apoptosis and migration induced by this combination, the targets and signaling pathways regulated by this combination, and the mechanisms of this combination’s synergistic effects on HeLa cells rather than on SiHa and C33A cells, etc. Moreover, our previous study demonstrated that PMGS down-regulated the expressions of the HPV-18 oncogene proteins E6 and E7 in Hela cells [11]. Additionally, HPV-18 is one of the main causes of cervical cancer [3,22]. Therefore, it is necessary to investigate whether PMGS or its combination with PTX plays an anti-tumor role in cervical cancer therapy through HPV-related carcinogenic mechanisms.

Our previous study has shown that PMGS treatment could elevate the cell densities of leukocytes and lymphocytes and promote the CD4^+^ T-cell population, which was reduced by carboplatin via the promotion of CD4^+^ T-cell differentiation and maturation and the inhibition of CD4^+^ T-cell apoptosis in mice [10]. These findings demonstrate that PMGS may play a dual role of enhancing effectiveness and reducing toxicity when combined with chemotherapy drugs. However, according to NCCN Guidelines, cisplatin/paclitaxel/bevacizumab and carboplatin/paclitaxel/bevacizumab are preferred regimens as first-line combination therapies [27]. Therefore, in order to obtain the best drug combination, we are still dedicated to studying the comprehensive effect of PMGS combined with other chemotherapy or antibody drugs in cervical cancer, such as cisplatin, carboplatin, and bevacizumab.

In conclusion, our study demonstrates that PMGS, a marine polysaccharide, combined with paclitaxel as a novel sensitizer, synergistically exerts anti-tumor effects on HeLa cervical cancer cells by enhancing cytotoxicity, inducing cell apoptosis, and inhibiting cell migration. It potentially provides a new combination medication for cervical cancer treatment and, thus, improves therapeutic efficacy.

## 4. Materials and Methods

### 4.1. Drugs

Marine sulfated polysaccharide PMGS with a content of sulfate of 36% and a molecular weight (Mw) of 153 kDa was prepared in our lab at the Marine Biomedical Research Institute of Qingdao. It was synthesized from marine alginate, which was purified from brown seaweed as described previously [10,11]. The chemical structure of PMGS is shown in Figure 1A. It was dissolved in a culture medium and prepared when used.

PTX was obtained from MedChemExpress (HY-B0015, Princeton, NJ, USA). The chemical structure of PMGS is shown in Figure 1B. As a stock solution, PTX was dissolved in DMSO and stored at −20°C. The working solution was prepared by dissolving the stock solution in a culture medium when used.

### 4.2. Cell Lines and Cell Culture

Human cervical cancer cell lines, HPV-18-positive Hela and HPV-negative C33A, were acquired from Procell Life Science&Technology Co., Ltd. (Wuhan, China), and HPV-16-positive SiHa was from Boster Biological Technology Co., Ltd. (Wuhan, China). The Hela cells were maintained in Dulbecco’s modified Eagle’s medium (DMEM, L110KJ, BasalMedia, Shanghai, China) supplemented with 10% fetal bovine serum (FBS, PWL001, Meilunbio, Dalian, China) and a 1% penicillin–streptomycin solution (PWL062, Meilunbio). The other cell lines were cultured in Minimum Essential Medium (MEM, L110KJ, BasalMedia) supplemented with 10% FBS and a 1% penicillin–streptomycin solution. The cell lines were maintained in an incubator at 37 °C with 5% CO_2_.

### 4.3. Cytotoxicity Assay

The cytotoxicity of PMGS or PTX in the Hela, SiHa, and C33A cells was determined using a Cell Counting Kit-8 (CCK-8) assay. Concisely, the cells (4 × 10^3^/well) were seeded into 96-well plates and treated with PMGS (0–20 mg/mL) or PTX (0–100 nM) for 48 h. Subsequently, a CCK-8 kit (MA0218, Meilunbio) was utilized to detect cell viability, in which cells were added to a medium containing 10% CCK-8 and further incubated for 2 h at 37 °C; then, the absorbance was measured using a multimode plate reader (VICTOR Nivo S, Perkin Elmer, Waltham, MA, USA) at 450 nm. The IC_50_ values were determined using GraphPad Prism software (version 8.0, GraphPad Software, La Jolla, CA, USA).

### 4.4. Drug Interaction Analysis

The Hela, SiHa, and C33A cells (4 × 10^3^/well) were treated with PMGS (0–22 mg/mL) or PTX (0–240 nM) alone or combined. The concentration gradient of PMGS or PTX was determined by IC_50_ values, which were calculated via the above cytotoxicity assay. After incubation for 48 h, a CCK8 assay was performed to identify cell viability as described above. Eventually, the online SynergyFinder software (https://synergy-finder.fimm.fi accessed on 11 April 2022) [28] was applied to estimate the drug interaction between PMGS and PTX. The synergy scoring was calculated utilizing the “viability readout” through the zero interaction potency (ZIP) calculation method [29]. ZIP synergy scores < −10 were considered to be antagonistic, scores from −10 to 10 were considered to be additive, and scores >10 were considered to be synergistic [29].

### 4.5. Colony Formation Assay

To detect cell proliferation after the different treatments, with either PTX (25 nM, 50 nM) or PMGS (2 mg/mL) alone or in combination, a colony formation assay was performed. The cells (2 × 10^3^/well) were seeded into 6-well plates; then, a fresh culture medium with the different treatments was added and replaced every 3 days for approximately 12 days until the colonies contained over 50 cells. The colonies were fixed with 4% paraformaldehyde (P1110, Solarbio, Beijing, China) and stained with 0.1% crystal violet (G1063, Solarbio). The colony formation number was quantified using ImageJ software (version 1.52a, National Institutes of Health, Bethesda, MD, USA).

### 4.6. Cell Apoptosis Analysis

An Annexin V-FITC/PI Apoptosis Detection Kit (40302ES60, Yeasen, Shanghai, China) was used to analyze the cell apoptosis percentage according to the manufacturer’s protocols. Briefly, after the different treatments, with either PMGS (2 mg/mL) or PTX (50 nM) alone or in combination for 48 h, the cells were digested by trypsin without EDTA (MA0234, Meilunbio), following incubation with Annexin V-FITC and propidium iodide (PI) for 15 min in the dark, and detected using a flow cytometer (CytoFlex, Beckman Coulter, Brea, CA, USA). FlowJo software (version 10.8.1, BD Bioscience, San Jose, CA, USA) was utilized to analyze the data.

### 4.7. Cell Cycle Analysis

The distribution of the cell cycle was determined utilizing a cell cycle detection kit (C1052, Beyotime, Shanghai, China) following the manufacturer’s instructions. In brief, after different treatments, with either PMGS (2 mg/mL) or PTX (50 nM) alone or simultaneously for 24 h, the cells were fixed with 70% ethanol overnight at 4 °C, then stained using PI solutions for 30 min at 37 °C in the dark, and finally detected using a flow cytometer (CytoFlex, Beckman Coulter). The results were analyzed using ModFit LT software (version 5.0, Verity Software House, Topsham, ME, USA).

### 4.8. Wound-Healing Assay

Cell migration was estimated via a wound-healing assay as described previously [30]. In short, the cells were seeded on 6-well plates and cultured until reaching confluence, and then a sterile 200 μL pipette tip was used to scrape a line in the cellular monolayer. After the cell debris was abolished, the cellular monolayer was incubated with either PMGS (2 mg/mL) or PTX (50 nM) alone or in combination for 24 h. Images were captured under an inverted microscope (ECLIPSE Ts2, Nikon, Tokyo, Japan) at 0 and 24 h after scratching. ImageJ software (version 1.52a, National Institutes of Health) was utilized to quantify the rate of wound closure.

### 4.9. Transwell Migration Assay

The migration of cells was also evaluated using a Transwell migration assay as described previously [30]. The cells (5 × 10^4^/well) were suspended in 200 μL of a serum-free medium containing PMGS (2 mg/mL) or PTX (50 nM) alone or in combination and were seeded into the upper chambers. Simultaneously, the lower chambers were added with 600 μL of media supplemented with 10% FBS as the attractant. After incubation for 48 h, the migrated cells on the lower chambers were fixed with 4% paraformaldehyde (P1110, Solarbio) and stained with 0.1% crystal violet (G1063, Solarbio). Finally, the migrated cells were captured and counted under an inverted microscope (ECLIPSE Ts2, Nikon).

### 4.10. Western Blot Assay

The cells were treated with either PMGS (2 mg/mL) or PTX (50 nM) alone or in combination for 48 h, and then the proteins were extracted using a cell lysis buffer (P0013, Beyotime) and subsequently subjected to a Western blot analysis as described previously [30]. Briefly, equal amounts of proteins, which were quantified using a BCA Protein Assay Kit (P0010, Beyotime), were electrophoresed by SDS–PAGE and transferred onto polyvinylidene fluoride (PVDF) membranes (IPVH00010, Merck Millipore, Billerica, MA, USA). The membranes were blocked utilizing Protein Free Rapid Blocking Buffer (PS108P, Epizyme, Shanghai, China), incubated with appropriate primary antibodies at 4 °C overnight, and then exposed to HRP-conjugated secondary antibodies (Proteintech, Wuhan, Hubei, China) for 1 h at room temperature. The signals were acquired on a ChemiDoc XRS+ imaging system (Universal Hood Ⅱ, Bio-Rad, Hercules, CA, USA) with Image Lab software. GAPDH was used as the internal control. The antibodies used in this study are listed in Table S1.

### 4.11. Statistical Analysis

All experiments and analyses were repeated at least three independent times. The results are presented as the mean ± standard error of the mean (SEM). All statistical analyses were performed using GraphPad Prism software (version 8.0, GraphPad Software, San Diego, CA, USA) and the online SynergyFinder software (version 3.0). The statistical significance of the differences was evaluated with Student’s *t* test (two groups) or a one-way analysis of variance (ANOVA) (more than two groups). Statistical significance was considered at * *p* value < 0.05; ** *p* value < 0.01; and *** *p* value < 0.001.

## Data Availability

The data supporting the findings of this study are available from the corresponding author upon reasonable request.

## Supplementary Materials

The following supporting information can be downloaded at https://www.mdpi.com/article/10.3390/md21050259/s1, Table S1: List of the antibodies used in this study; Table S2: Dose range for drug combination analysis in Hela cells; Table S3: Dose range for drug combination analysis in SiHa cells; Table S4: Dose range for drug combination analysis in C33A cells.
