# Supplementary material for: Marine Sulfated Polysaccharide PMGS Synergizes with Paclitaxel in Inhibiting Cervical Cancer In Vitro
Source: Mar Drugs. 2023 Apr 23;21(5):259. doi: 10.3390/md21050259 (PMC10221832; doi:10.3390/md21050259)
Supplement: Supplementary file 1 [file marinedrugs-21-00259-s001.zip › marinedrugs-2355510-supplementary.pdf]

Supplementary Tables

Table S1. List of the antibodies used in this study

Name	Catalog number	Source
Caspase-3	9662	Cell Signaling Technology
Caspase-9	9502	Cell Signaling Technology
GAPDH	60004-1-Ig	Proteintech

Table S2. Dose-range for drug combination analysis in Hela cells

		PMGS (mg/mL)					
		**0**	**1**	**2**	**4**	**8**	**16**
PTX (nM)	**0**	100.00	82.47	82.89	79.72	76.97	57.37
	**6.25**	78.34	67.09	62.56	60.08	53.76	43.26
	**12.5**	69.18	49.20	49.26	46.49	39.61	34.77
	**25**	61.33	42.70	41.11	40.13	35.20	30.71
	**50**	54.15	30.31	30.03	29.31	26.29	25.06
	**100**	52.04	27.97	27.59	27.81	26.37	22.57

Hela cells were treated either with PMGS (0-16 mg/mL) or PTX (0-100 nM) alone or in combination for 48 hrs. Then, cell viability was determined by a CCK-8 assay and shown as above. Eventually, the result was uploaded to the online SynergyFinder software (https://synergy-finder.fimm.fi) to identify the drug interaction between PMGS and PTX using zero interaction potency (ZIP) synergy scores[1, 2].

Table S3. Dose-range for drug combination analysis in SiHa cells

		PMGS (mg/mL)					
		**0**	**1.375**	**2.75**	**5.5**	**11**	**22**
**PTX (nM)**	**0**	100.00	118.39	97.69	105.05	71.13	64.44
	**15**	79.91	82.99	69.05	66.05	51.69	49.25
	**30**	81.81	83.34	72.16	60.21	49.89	47.19
	**60**	75.37	75.49	67.30	66.41	48.01	35.31
	**120**	60.13	62.06	57.94	38.47	39.69	18.72
	**240**	60.11	61.29	55.40	49.98	40.79	37.45

SiHa cells were treated either with PMGS (0-22 mg/mL) or PTX (0-240 nM) alone or in combination for 48 hrs. Then, cell viability was determined by a CCK-8 assay and shown as above. Eventually, the result was uploaded to the online SynergyFinder software (https://synergy-finder.fimm.fi) to identify the drug interaction between PMGS and PTX using zero interaction potency (ZIP) synergy scores[1, 2].

Table S4. Dose-range for drug combination analysis in C33A cells

		PMGS (mg/mL)					
		**0**	**1.375**	**2.75**	**5.5**	**11**	**22**
**PTX (nM)**	**0**	100.00	129.51	128.95	109.03	83.36	64.73
	**15**	89.32	94.71	89.33	87.16	81.92	65.30
	**30**	92.41	96.20	96.21	82.07	67.58	57.63
	**60**	85.40	86.05	86.63	83.46	69.12	43.93
	**120**	87.39	87.18	87.84	70.75	57.35	44.36
	**240**	78.78	94.33	90.23	82.02	65.23	45.98

C33A cells were treated either with PMGS (0-22 mg/mL) or PTX (0-240 nM) alone or in combination for 48 hrs. Then, cell viability was determined by a CCK-8 assay and shown as above. Eventually, the result was uploaded to the online SynergyFinder software (https://synergy-finder.fimm.fi) to identify the drug interaction between PMGS and PTX using zero interaction potency (ZIP) synergy scores[1, 2].

References

[1] Ianevski A., He L., Aittokallio T., et al. SynergyFinder: a web application for analyzing drug combination dose-response matrix data. Bioinformatics, 2017, 33(15): 2413-2415.

[2] Ianevski A., Giri A.K., and Aittokallio T. SynergyFinder 3.0: an interactive analysis and consensus interpretation of multi-drug synergies across multiple samples. Nucleic Acids Research, 2022, 50(W1): W739-W743.
